# SPIRITUALITY IN CHRONIC PAIN SELF-MANAGEMENT: LOWER USE BUT EQUAL IMPORTANCE IN OLDER AFRICAN AMERICANS

**DOI:** 10.1093/geroni/igz038.1452

**Published:** 2019-11-08

**Authors:** Staja Booker

**Affiliations:** The University of Florida, Gainesville, Florida, United States

## Abstract

Spirituality is a key social determinant of health for African Americans (AAs) and strongly impacts management of chronic pain. Older AAs (average age 68± 12.37) from urban and rural communities completed questionnaires (N= 110) and audio-recorded, semi-structured individual interviews (N= 18) describing osteoarthritis pain self-management. Prayer was used by 42% of AAs, with substantially fewer attending church (23.6%), watching religious television or reading the Bible/Christian literature (20.9%), listening to gospel music (18.2%), and laying of hands (8.2%). Interestingly, prayer and church attendance were the only pain strategies rated by more participants as very helpful. Regardless of religiosity, most AAs believed that spirituality was “an important aspect, whether we realize it always or not”. Specifically, prayer was considered “number one… ‘cause I know it’s gonna be all right once I do pray…prayer help heal the pain”. Spiritual strategies remain integral for chronic pain self-management despite lower than expected use among AAs.

